# Remote sensing image description based on word embedding and end-to-end deep learning

**DOI:** 10.1038/s41598-021-82704-4

**Published:** 2021-02-04

**Authors:** Yuan Wang, Hongbing Ma, Kuerban Alifu, Yalong Lv

**Affiliations:** 1 Department of College of Information Science and Engineering, Xinjiang University, Urumqi, China; 2 Department of Electronic Engineering, Tsinghua University, Beijing, China; 3 Department of College of Software, Xinjiang University, Urumqi, China

**Keywords:** Computer science, Information technology

## Abstract

This study proposes an end-to-end image description generation model based on word embedding technology to realise the classification and identification of *Populus euphratica* and Tamarix in complex remote sensing images by providing descriptions in precise and concise natural sentences. First, category ambiguity over large-scale regions in remote sensing images is addressed by introducing the co-occurrence matrix and global vectors for word representation to generate the word vector features of the object to be identified. Second, a new multi-level end-to-end model is employed to further describe the content of remote sensing images and to better advance the description tasks for *P. euphratica* and Tamarix in remote sensing images. Experimental results reveal that the natural language sentences generated using this method can better describe *P. euphratica* and Tamarix in remote sensing images compared with conventional deep learning methods.

## Introduction

The development of satellite and remote sensing technologies in recent years has generated widespread interest in the applications of remote sensing images. Most current remote sensing image research focuses on target recognition^1^, image segmentation^2^, and scene classification^3^. While these studies can effectively identify objects of interest and obtain the class label of the object type in remote sensing images, the relation between the attributes of the object to be identified and the object itself is ignored. Establishing this relation would require describing the contents of an image.

To address this issue, researchers have designed and implemented many methods for the description and representation of images. Zhang^4^ proposed bag of visual words and multi-layer clustering analysis to resolve the semantic gap between objects to be recognised in remote sensing images. The retrieval performance of the algorithm was improved by combining a sparse automatic encoder with convolutional neural networks (CNNs), which reduced the time required for labelling and improved the operational efficiency of the model. Ghamisi^5^ applied conventional machine learning and one-dimensional (1-D) CNNs to classify remote sensing images. While this resulted in information loss in the form of hyperspectral pixels, a sequence recursive neural network image representation framework was proposed, which was inferred through a novel RNN model to determine the category of the information. Prasad^6^ regarded hyperspectral image data as a spectral sequence and employed a recurrent neural network (RNN) to simulate the dependence between different spectral bands. The underlying contextual semantic information was then obtained from the sequence using a convolutional recursive neural network. Alom^7^ adopted the recursive CNN (RCNN) and recursive residual CNN (RRCNN) of U-Net to propose a feature-accumulated depth framework for natural image semantic segmentation.The viability of the approach was demonstrated experimentally using medical images. Cai^8^ proposed a stacked CNN-RNN model end-to-end consisting convolutional long short-term memory (CLSTM) units in both top-down and bottom-up directions, which regularizes the segmentation of an image by integrating predictions of its neighboring slices. The model was employed to detect variability in pancreas images and avoid discontinuities at the pancreas boundary owing to the segmentation process. Zhen^9^ proposed a new heterogeneous simplified pulse coupled neural network model to segment greyscale images into several regions; the effectiveness of the segmentation was tested using images of actual cerebral cortex structures. Wei^10^ proposed a multi-direction text detection method to address the complex changes in perspective and scale direction in natural images; a deep CNN model was adopted to trim the boundaries between different objects and filter the non-character regions in images. Anderson^11^ employed a cellular simultaneous recurring network to conduct the training process for image representation, initialise the parameter generation target image, store the processed sub-image and realise the representation task of the image transformation. Byeon^12^ adopted a long short-term memory (LSTM) network to address class label ambiguities in the pixel-level segmentation and scene classification of images. The approach not only considered the complex spatial dependence of labels but also effectively learned the textural and spatial characteristics of images. Li^13^ proposed a deep image detection and description framework by combining enhanced learning with a deep CNN to address the increased difficulty of aircraft identification in remote sensing images owing to a series of problems such as illumination and changes in aircraft type and size. The approach was demonstrated to accurately identify the specific positions of aircraft in remote sensing images. Qu^14^ proposed a deep multimodal neural network model to describe the semantic information of objects of interest in remote sensing images. The validity of the approach was demonstrated, revealing that the model could effectively extract the semantic information of objects of interest and better describe the contents of remote sensing images. Scarpa^15^ designed a very compact architecture using a CNN to achieve precise training of small-sized data sets; a good recognition effect was obtained for images derived from various multi-resolution sensors. Maggiori^16^ proposed a spatially fine classification algorithm based on the pixel semantics of images obtained from aeronautical satellites in conjunction with a deep CNN. The output of the CNN model was improved, which enhanced its classification performance. Geng^17^ first proposed a new depth-supervised and compressed neural network to address the issues associated with the presence of speckle noise in synthetic aperture radar images and the lack of effective features to characterise images. Liu^18^ adopted a deep learning method to classify hyperspectral images. Two convolutional nerves were connected in parallel, and a deep transfer learning algorithm was proposed for local image description. Subsequently, deep features were extracted from each band and input into a recurrent neural network to realise image description and classification. However, the performance of the model was poor owing to the lack of training samples. Lu^19^ introduced a coding and decoding framework to translate images into natural sentences, which effectively resolved scale ambiguity and rotational ambiguity and obtained a clear reference data set (RSICD).

The aforementioned research has greatly advanced the application of semantic information to the higher-level comprehension of the scenes in images. However, the sentences generated by the above studies are simple natural language sentences that cannot effectively describe the content of complex remote sensing images and are typically employed in conjunction with small-scale data sets. Therefore, a new end-to-end model is constructed herein to decode remote sensing images into natural language sentences and complete the task of remote sensing image description. The proposed method is experimentally verified by its application to the classification and identification of the species *Populus euphratica* and Tamarix in complex QuickBird remote sensing images and unmanned aerial vehicle (UAV) images, both of which contain partially fuzzy image attributes. In addition, the description ability and recognition effect of the proposed method are compared with those obtained using conventional deep learning models. In order to further evaluate the generalization ability of the model, the End-to-end model was used to make relevant analysis in the public data set. The experimental results demonstrate the feasibility of the proposed method.

## Image representation

### Word vector representation

This study takes advantage of the correlation between adjacent pixels in remote sensing images and employs the global vectors for word representation (GloVe) model to mine the contextual semantic information of pixels in the neighbourhood window. Then, each word is mapped into a low-dimensional vector space and the similarities between the various pixels in a remote sensing image are calculated from the obtained word vector features.

Suppose that *x*_*i*,*k*_ represents the number of occurrences of pixel *k* and pixel *i* in a fixed window when training the word vector library. The sum of pixels *x*_*i*_ in a neighbourhood window is then given as follows:

1 $$ x_{i} = \sum\limits_{k = 1}^{m} {x_{i,k} } . $$

where *m* represents the total number of pixels in an appropriate dictionary. The probability of pixel *k* appearing in a fixed window is given as

2 $$ p_{ik} = \frac{{x_{i,k} }}{{x_{i} }}. $$

We then calculate the ratio of probabilities for pixels *i*, *j* and *k* as follows:

3 $$ R_{i,j,k} = \frac{{p_{i,k} }}{{p_{j,k} }}. $$

Among them, the value of R is close to 1, indicating that the pixels *i *and *k* are correlated, and the pixel *j* and *k* are not correlated, and vice versa. The original probability R can better distinguish the related pixels from the unrelated pixels, and can better distinguish the two related pixels in the region.

Because the co-occurrence relation between pixels is unbalanced, pixels with an unreasonable co-occurrence relation will be given minimal weight when the model parameters are running. Therefore, we introduce the weight equation $f(x_{i,j} )$ to address this issue. The objective function is given as follows:

4 $$ J_{\theta } = \sum\limits_{i,j = 1}^{m} {f(x_{i,j} )(\omega_{i}^{T} \tilde{\omega }_{j} + b_{i} + \overset{\lower0.5em\hbox{$\smash{\scriptscriptstyle\frown}$}}{b}_{j} - \log (x_{ij} ))^{2} } , $$

where $$\omega_{i}^{T}$$ represents the transpose of the word vector of pixel *i* when $$\omega_{i}$$ is the context and $$b_{i} ,\overset{\lower0.5em\hbox{$\smash{\scriptscriptstyle\frown}$}}{b}_{j}$$ represent the bias values.

**Figure 1:**
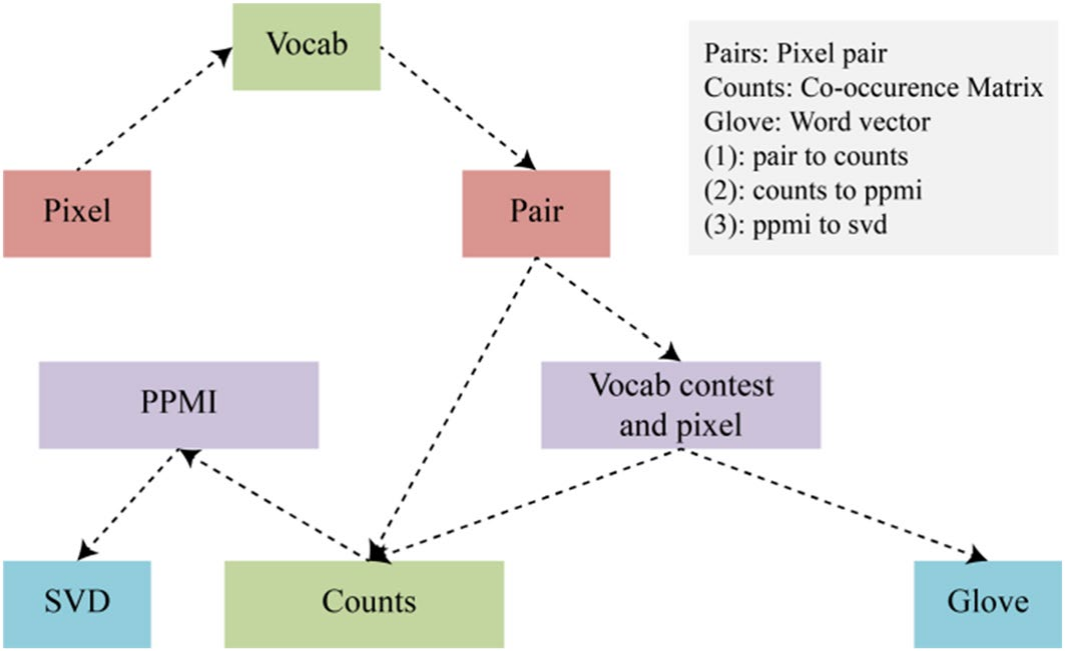
Network structure of GloVe and the co-occurrence matrix.

The structure of the co-occurrence matrix and GloVe word vector network is shown in Fig. 1. In addition, the co-occurrence matrix further describes the semantic relevance between adjacent pixels while describing the pixels of interest in a remote sensing image.

### Image labelling strategy

**Figure 2:**
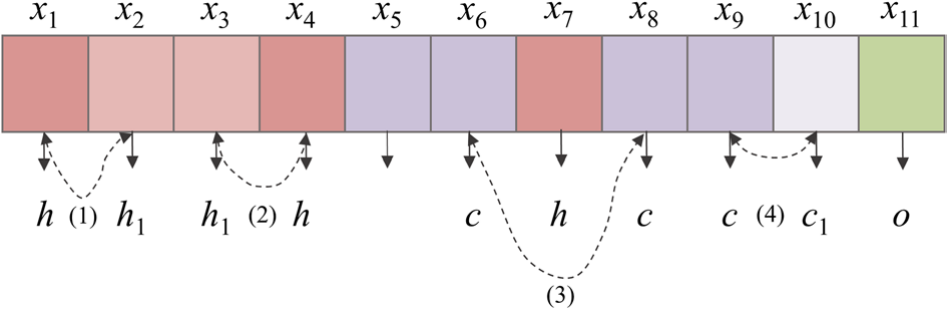
Example of pixels *x*_*i*_ (*i* = 1–11) and pixel types *h*, *c* and *o* illustrating the proposed labelling strategy. The points (1)–(4) represent transformations of pixel types in the imaging process.

To better describe the content of remote sensing images, the correlation between adjacent pixels is further explored using a new proposed labelling strategy. A pixel tagging example is shown in Fig. 2.

In Fig. 2, $x = \{ x_{1} ,x_{2} , \ldots ,x_{n} \}$ represents all the pixels in the remote sensing image; h, c and o represent labels for different types of pixels, namely those representative of *P. euphratica*, Tamarix and other objects. h1 and c1 represent pixels with similar characteristics to those of h and c, respectively. Due to the influence of the environment or the sensor itself, pixels of equivalent types are transformed into different types in the imaging process, which is indicated by points (1), (2) and (4) in Fig. 2. Point (3) represents a condition wherein a non-homogeneous pixel lies between two pixels of equivalent type, which can then be considered as being the same pixel type as that of the surrounding pixels. Otherwise, a pixel is treated according to its original type. Therefore, this process is equivalent to conducting a multi-label serialisation process involving fine-grained classification based on multiple labels. The proposed labelling strategy is always fixed to a window comprising 10 pixels. The decoder is composed of an LSTM network with a deviation loss function and IndRNN an independently recurrent neural network (IndRNN)^20^, which enhances the correlation between adjacent pixels and labels by eliminating the gradient fading problem.

## End-to-end model

**Figure 3:**
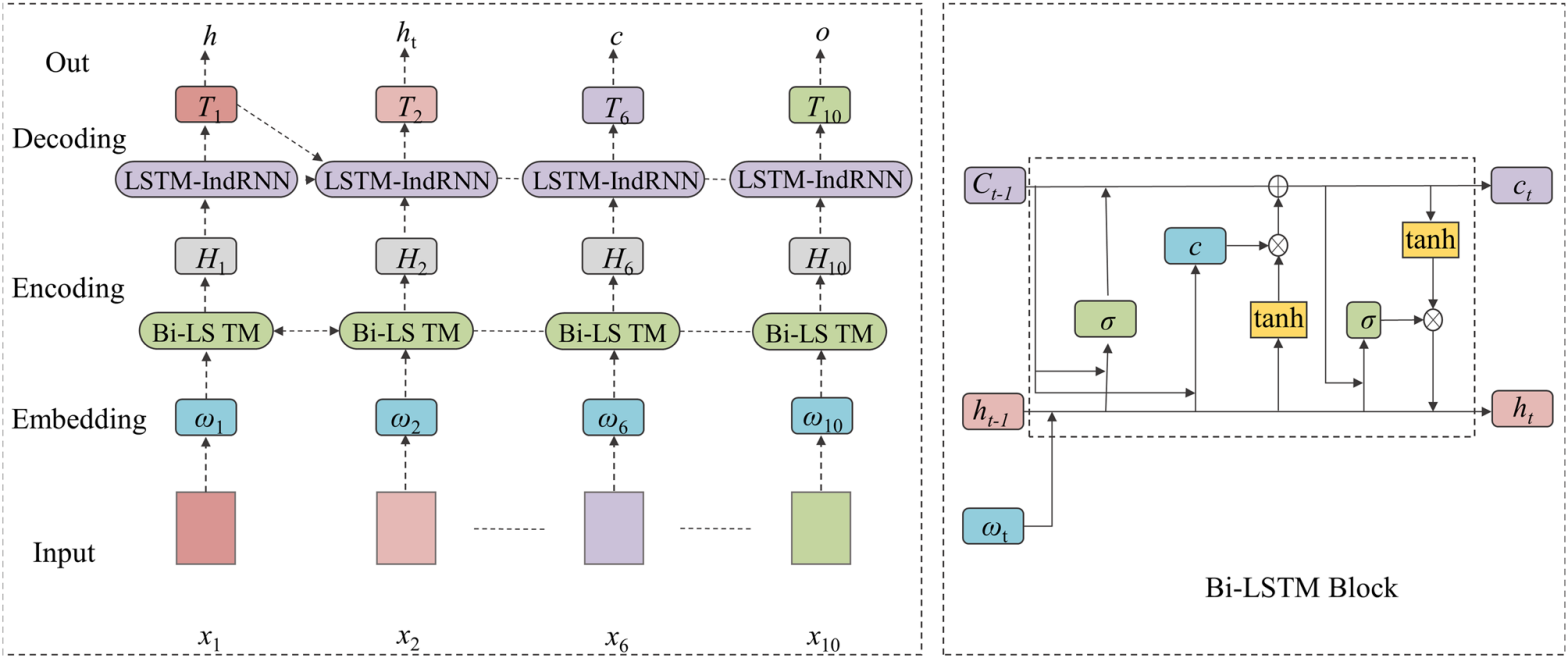
Network structure of the end-to-end model.

In recent years, end-to-end models based on neural networks^21^ ^22^ have been widely used for tagging tasks in natural language sequence processing; this study employs this model to generate label sequences. As shown in Fig. 3, the model is composed of an embedding layer and two basic layers: encoding and decoding. The embedding layer maps labels in a low-dimensional idea vector space to extract the relation between labels and 1pixels. The coding in the coding layer is conducted using a bidirectional LSTM network (Bi-LSTM)^23^. In the sequence labelling process, the Bi-LSTM coding layer can capture the detailed information of each pixel and obtain the global semantic information of a remote sensing image. Finally, the decoding layer employs an LSTM network and IndRNN decoding structure to generate label sequences to further extract the relation between labels and pixels.

After the pixels in the remote sensing image are mapped according to word vectors, two parallel LSTM layers, denoted as the forward LSTM layer and the backward LSTM layer, are stored at each time step *t* in a set of cyclically linked subnets. The LSTM^24^ storage block in the Bi-LSTM coding layer calculates the current hidden vector $h_{t}$ and current unit vector ct from their previous values at *t* − 1, as indicated by the network structure shown in the Bi-LSTM block of Fig. 3. The specific calculations of the terms in Fig. 3 at the present time step t are given as follows.

5 $$ i_{t} = \lambda (\theta_{\omega i} x_{t} + \theta_{hi} h_{t - 1} + \theta_{ci} c_{t - 1} + b_{i} ), $$

6 $$ f_{t} = \lambda (\theta_{\omega f} x_{t} + \theta_{hf} h_{t - 1} + \theta_{cf} c_{t - 1} + b_{f} ), $$

7 $$ z_{t} = \tanh (\theta_{\omega c} x_{t} + \theta_{hc} h_{t - 1} + b_{c} ), $$

8 $$ c_{t} = f_{t} c_{t - 1} + i_{t} z_{t} , $$

9 $$ o_{t} = \lambda (\theta_{\omega o} x_{t} + \theta_{ho} h_{t - 1} + \theta_{co} c_{t - 1} + b_{o} ), $$

10 $$ h_{t} = o_{t} \tanh (c_{t} ), $$

where *i* represents the input gate, the terms involving *θ* represent the corresponding parameters, *x* represents the current pixel, the terms involving *b* represent the corresponding bias unit, *f* represents the forgetting gate and *o* represents the output gate.

**Figure 4:**
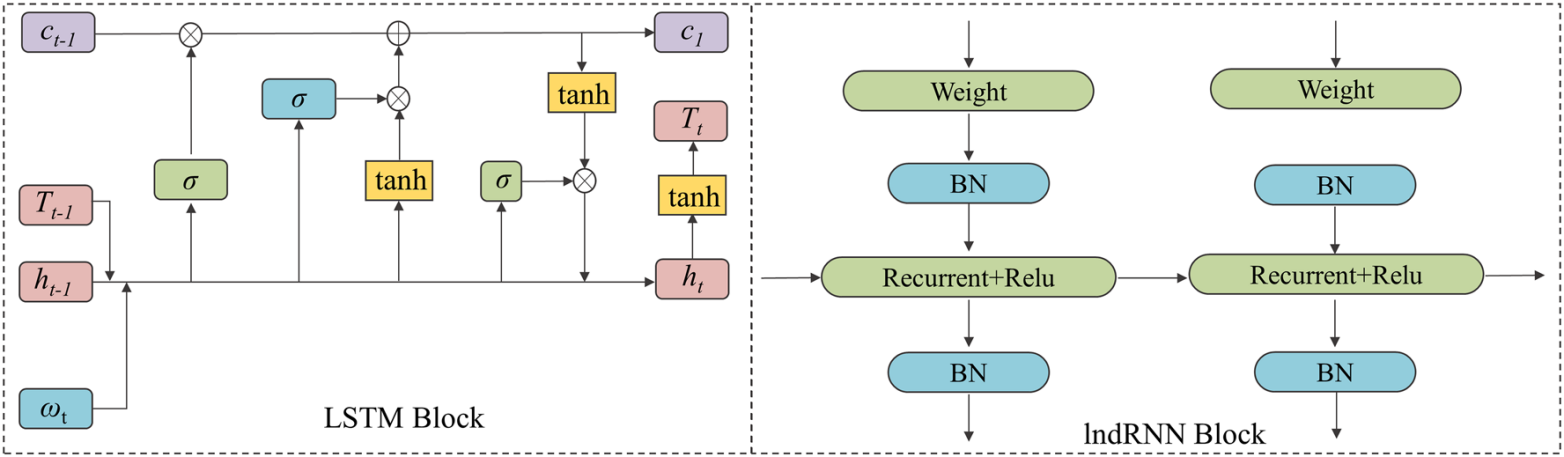
Network structure of the LSTM block and the IndRNN block of the decoding module.

During decoding, the input of the decoding layer is *h*_*t*_ when the label of pixel *x*_*t*_ is detected and an embedded prediction label *T*_*t*−1_ is obtained from the Bi-LSTM encoding layer. The partial calculation performed by the IndRNN is given as follows:

11 $$ h_{t} = \lambda (\theta x_{t} + \upsilon \odot h_{t - 1} + b), $$

12 $$  h_{{n,t}}  = \lambda (\theta _{n} x_{t}  + \upsilon _{n} h_{{n,t - 1}}  + b_{n} )  $$

here *θ*, *ν* and e represent the input weight, loop weight and Adarma product, respectively, where *n* represents the current values of line n. In an IndRNN, each neurone is independent of each other. As such, each neurone only receives the information of the current state hidden layer and the input layer and independently aggregates the spatial information of the image with respect to the time steps. The specific structure of the LSTM network and IndRNN block is illustrated in Fig. 4.

A softmax classifier is employed to calculate the normalised pixel label probability based on the pixel label prediction vector $T_{t}$ as follows.

13 $$ y_{t} = \theta_{y} T_{t} + b_{y} , $$

14 $$ p_{t}^{i} = \frac{{e^{{y_{t}^{i} }} }}{{\sum\limits_{k = 1}^{{N_{t} }} {e^{{y_{t}^{i} }} } }}. $$

here *θ*_*y*_ represents the matrix and *N*_*t*_ is the number of labels.

Finally, the model is optimised using the maximum log likelihood function RMS-Sprop^25^.

## Experimental results and analysis

### Experimental setup

**Figure 5:**
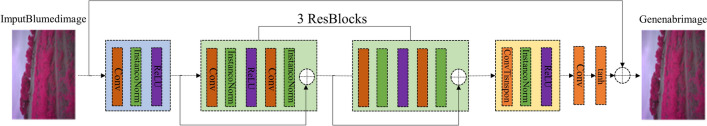
Structure of the generative adversarial network (GAN)^28,29^ employed to sharpen blurred images.

The performance of the proposed method in terms of feature representation and image description was evaluated for describing *P. euphratica* and *T. ramosissima* using UAV remote sensing images obtained over southern Luntai County, Xinjiang Uygur Autonomous Region as the experimental dataset along with high-resolution QuickBird satellite images. The UAV images suffered from an adjustment problem when capturing the images. Therefore, a part of the images were blurred. To address this issue, a generative adversarial network (GAN)^26,27^ was employed to sharpen the obtained images. The specific GAN structure is illustrated in Fig. 5.

In Fig. 5, the sizes of the Conv cores from left to right are 128 × 5 × 5, 256 × 3 × 3 and 128 × 5 × 5, respectively. The $\oplus$ module represents the residual network connexion block. The GAN is primarily based on the antagonism of ResNet blocks^30^. Its input is the original blurred image rather than noise data. The GAN can not only reduce blurring but also strengthen the characterisation ability of P. euphratica and T. ramosissima pixels.

### Evaluation criteria and model initialisation parameters

The precision (P), recall (R) and F-score values were employed as the evaluation criteria. The F-score value is calculated as follows:

15 $$ F = \frac{2 * P * R}{{P + R}}. $$

Compared with conventional pixel-by-pixel recognition methods, the proposed method uses the adjacent pixels of the same column or adjacent pixels in a fixed length of the same row as the input of the end-to-end model and the labels adopt an equivalent embedded mapping based on label serialisation annotation.

The values of the model parameters can affect the overall performance of the model and affect the characterisation ability of the word vector features. Therefore, the parameter settings play a vital role in the performance of the model. Accordingly, we adjusted the variable parameters to achieve an optimal model performance. The initial model parameters are listed in Table 1.

**Table 1:** Parameter settings.

End-to-end Model	
**Encoding-Bi-LSTM**	
Neural unit	150
Dropout	0.5
**Decoding-LSTM**	
Neural unit	200
Dropout	0.25
**Decoding-IndRNN**	
Neural unit	32/64
Dropout	0.25
Recurrent dropout	0
**End-to-end**	
Learning rate	0.001
Optimizer	RMS

Table 1 represents the basic parameter settings; however, the specific parameters employed depend on the actual conditions. The number of neurones in the Bi-LSTM encoding layer varies according to the word vector dimension i.e. 150, 200 and 100, whereas the number of LSTM nerve units in the decoding layer of the first layer is doubled with the coding.

### Analysis of experimental results

The training was conducted using 20% of the test set. The experimental verification work was conducted to investigate the description ability of the model under the following conditions: (1) the effect of the number of coding and decoding layers, (2) the effect of adopting different network models in the coding and decoding layers, e.g. replacing the Bi-LSTM network with the IndRNN, (3) the effect of the word vector dimension, (4) compared with a conventional labelling method, (5) the effect of the model on the public data set (6) compared with conventional depth learning models.

#### Number of coding and decoding layers

**Figure 6:**
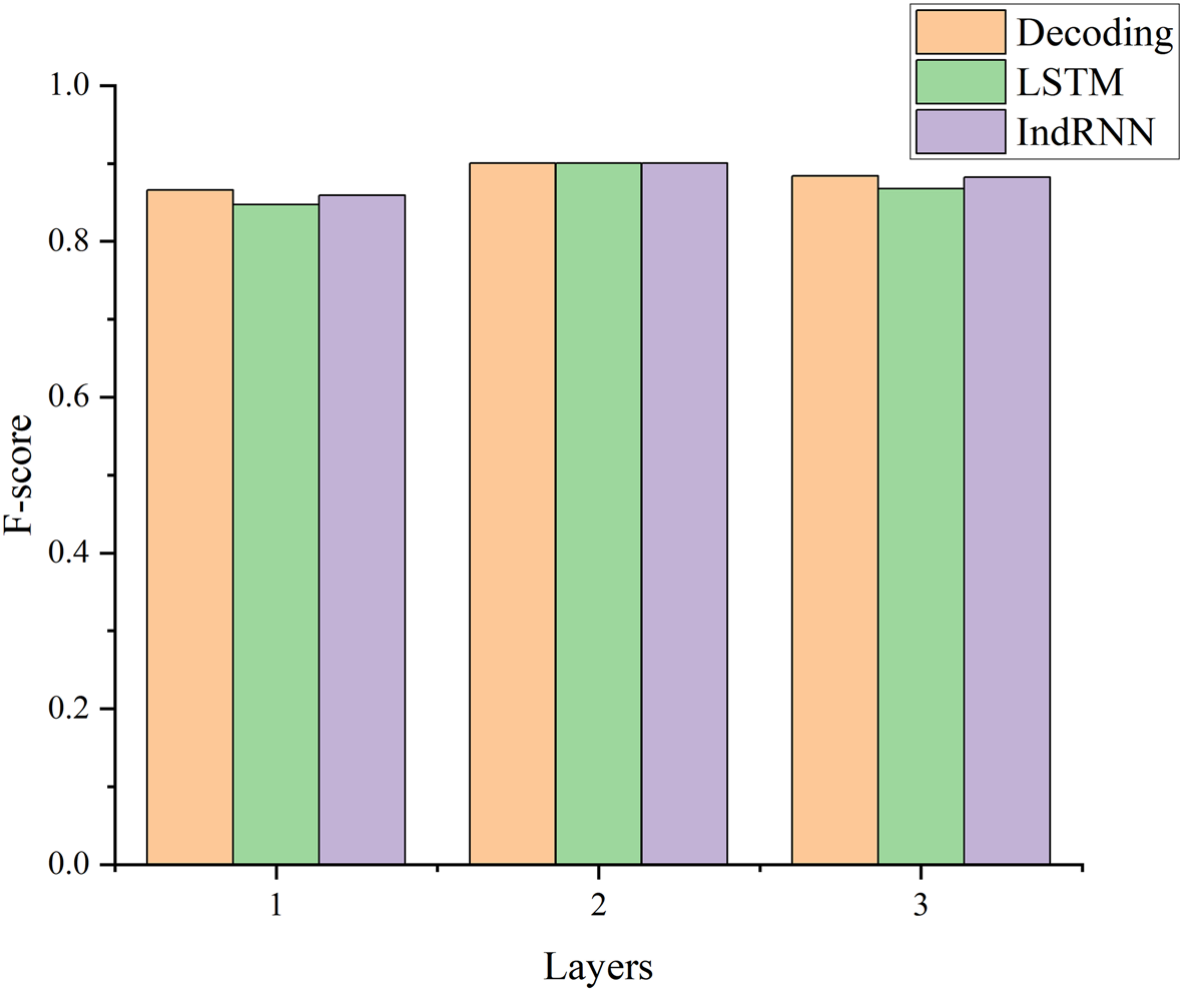
Effect of the number of coding and decoding layers on the F-score.

Overfitting may occur during the training process when the number of layers is considerably large. Thus, the model tends to become trapped in local optima. However, the model cannot learn hidden features if few layers are adopted. Therefore, we investigated the effect of the number of decoding and coding layers on the P, R and F criteria values. The experimental F-score results are shown in Fig. 6. P, R and F criteria values are listed in Table 2.

**Table 2:** Effect of the number of coding and decoding layers on the evaluation criteria values.

Number of layers—percentage	Decoding	LSTM	IndRNN
**1**			
P	0.8712	0.8594	0.8632
R	0.8601	0.8359	0.8467
F	0.8656	0.8474	0.8593
**2**			
P	0.9146	0.9146	0.9146
R	0.8861	0.8861	0.8861
F	0.9001	0.9001	0.9001
**3**			
P	0.8934	0.8837	0.8903
R	0.8749	0.8519	0.8739
F	0.8840	0.8675	0.8820

The results in Fig. 6 and Table 2 exhibit the following. The decoding in the above chart indicates that both the LSTM layer and the IndRNN layer increase and decrease simultaneously, while the LSTM below indicates that the IndRNN layer is fixed at two layers, whereas the IndRNN layer is fixed at two layers when the IndRNN layer increases and decreases. The forward and backward LSTM layers in the coding layer are characterised by the word vector (GloVe).

##### Analysis

1. Table 2 reflects that the end-to-end model obtains the highest F-score value of 0.9001 indicative of its best ability to characterise the features of images when the coding layer employs the Bi-LSTM network and the decoding layer employs an LSTM network and IndRNN that are both two layers.
2. The decoding effect decreases as the number of decoding layers increases, and overfitting may occur. Therefore, the model fails to learn the hidden features effectively, resulting in a poor feature representation ability.
3. As discussed, each neurone in the IndRNN is not only independent from all other neurones, but can also effectively solve the problem of gradient disappearance during long time series learning. Therefore, the feature characterisation ability of the IndRNN is better than that of the LSTM network.

#### Substitution of coding and decoding layer network models

**Figure 7:**
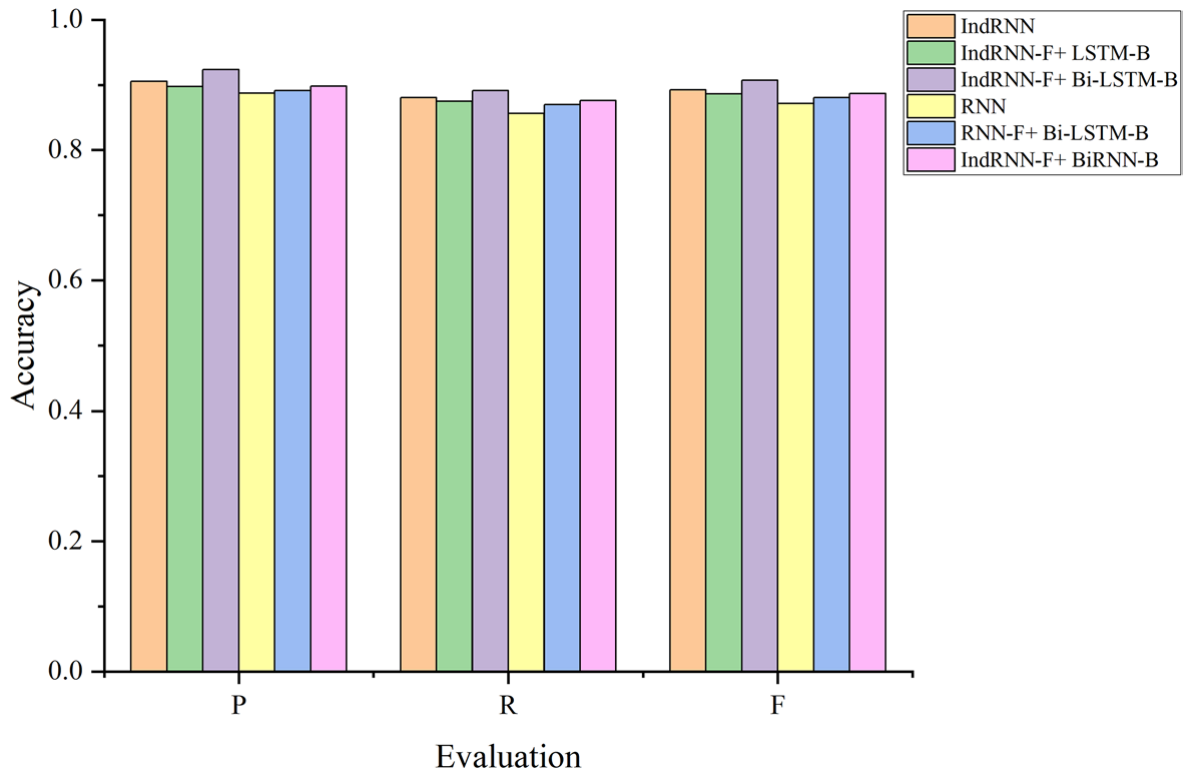
Effect of various coding and decoding layer network models on the F-score.

The ability of different network models to learn features is not uniform. Therefore, we adopted different network models in the coding and decoding layers and evaluated the resulting description performance on the experimental data sets. The experimental F-score results are shown in Fig. 7; all P, R and F criteria values are listed in Table 3. In the table, the affixes -F and -B represent the forward and backward directions, respectively. Both the Bi-LSTM network and IndRNN in the decoding layer employ two layers in the experiments.

1. The optimum F-score value of 0.9069 at Table 3 is obtained when the forward propagation of the coding layer is the IndRNN and the backward propagation is the Bi-LSTM network. This is because the neurons in the IndRNN are independent and facilitate the cross-layer transmission of information, which can better learn hidden details.
2. Compared with the LSTM network in Fig. 7, the performance of the RNN is not satisfactory because the RNN model loses valid information when the input sequence is too long; therefore, it is not well suited for representing the content of remote sensing images.
3. The IndRNN-F + LSTM-B combination provides smaller P, R and F values than the IndRNN-F + BiRNN-B combination because a single LSTM network can learn long sequences effectively; however, it ignores semantic information between some of the pixels in the fixed window and the global image. As such, it is not well suited for describing image contents.

**Table 3:** Effect of various coding and decoding layer network models on the evaluation criteria values.

Percentage—decoding layer	P	R	F
IndRNN	0.9053	0.8803	0.8926
IndRNN-F + LSTM-B	0.8976	0.8751	0.8862
IndRNN-F + Bi-LSTM-B	0.9234	0.8911	0.9069
RNN	0.8871	0.8562	0.8713
RNN-F + Bi-LSTM-B	0.8914	0.8695	0.8803
IndRNN-F + BiRNN-B	0.8981	0.8759	0.8868

#### Word vector dimensions

**Figure 8:**
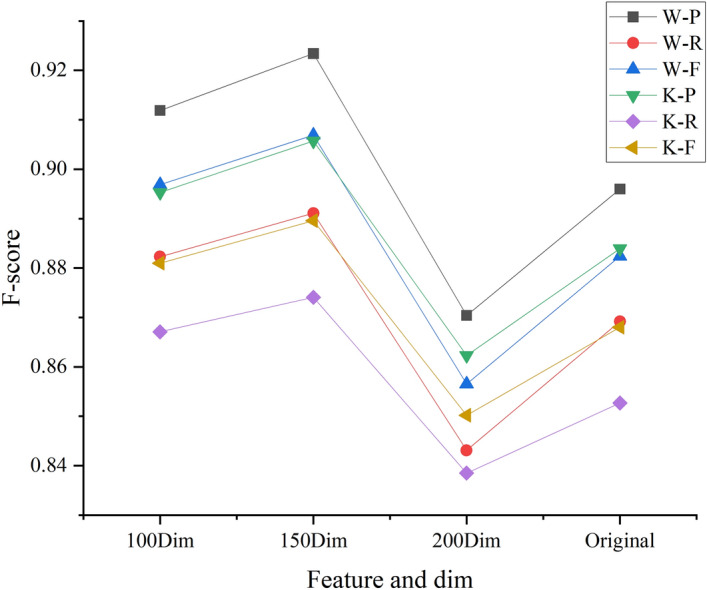
Effect of the different word vector dimensions of different sensors on the F-score.

The experimental F-score results obtained for the different word vector dimensions of different sensors are shown in Fig. 8; P, R and F criteria values are listed in Table 4. In Fig. 8, w–p represents the remote sensing image accuracy of the UAV and k-p represents QuickBird remote sensing image data. Original features of Original generation images.

**Table 4:** Effect of the different word vector dimensions of different sensors on the evaluation criteria values.

Percentage—decoding layer	100Dim	150Dim	200Dim	Original
**UAV**				
P	0.9119	0.9234	0.8704	0.8960
R	0.8823	0.8911	0.8431	0.8692
F	0.8969	0.9069	0.8565	0.8824
**Quick Bird**				
P	0.8953	0.9057	0.8623	0.8839
R	0.8671	0.8741	0.8385	0.8527
F	0.8810	0.8896	0.8502	0.8680

##### Analysis

1. The word vector dimension is 150, regardless of UAV image or Quick Bird image, the recognition accuracy and F-score value are the best.The representation space becomes increasingly sparse as the word vector dimension increases, and the semantic information of the original pixel cannot be well described when the word vector is small.
2. The representation ability of the word vector is better than the original pixel characteristic. The original features only express the characteristics of a single pixel and cannot mine the correlation between image pixels. The word vector feature also learns the global semantic information of the image while learning the correlation between adjacent pixels. Therefore, the word vector has a greater ability to describe an image than a single original characteristic.
3. Although QuickBird remote sensing images contain abundant spectral characteristics, they suffer from low imaging accuracy and a large number of spatial characteristics are neglected during image transmission because of the influence of the high altitude environment. Therefore, the UAV remote sensing image recognition accuracy and F-score values are greater than those obtained for QuickBird remote sensing images from Fig. 8.

#### Comparison with a conventional labelling method

**Figure 9:**
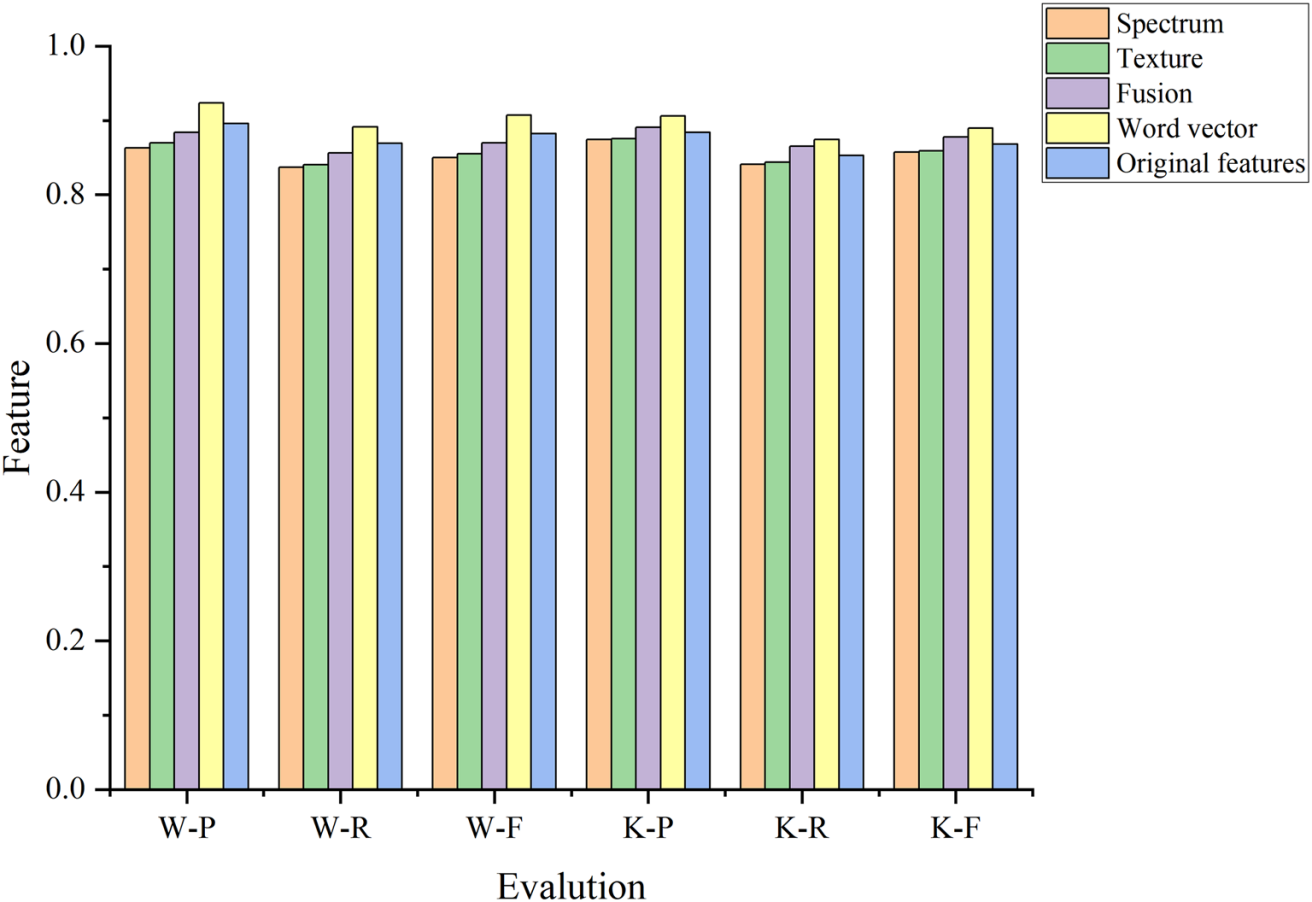
Labelling results of the proposed annotation strategy and a conventional pixel-by-pixel annotation method.

Here, we compare the proposed annotation strategy with a conventional pixel-by-pixel annotation method. In the conventional labelling method, the characteristics are divided into spectrum, texture and fused spectrum-texture. The vector labels are 01 (*P. euphratica*), 10 (Tamarix) and 11 (others). The experimental F-score results are shown in Fig. 9, and all P, R and F criteria values are listed in Table 5. In the figure, W represents UAV images, K represents QuickBird remote sensing image data, Spectral represents spectral features, Texture represents texture features, Fusion represents fused spectral-texture features, Word represents word vector features and Original represents original texture features.

**Table 5:** Effect of the different labelling methods on the evaluation criteria values.

Percentage—decoding layer	Spectrum	Texture	Fusion	Word vector	Original features
**UAV**					
P	0.8631	0.8699	0.8837	0.9234	0.8960
R	0.8372	0.8402	0.8563	0.8911	0.8692
F	0.8499	0.8548	0.8698	0.9069	0.8824
**Quick Bird**					
P	0.8743	0.8755	0.8905	0.9057	0.8839
R	0.8411	0.8436	0.8654	0.8741	0.8527
F	0.8574	0.8593	0.8778	0.8896	0.8680

##### Analysis

1. The proposed annotation strategy provides superior P, R and F values to the conventional scheme because the conventional method adopts single pixels for labelling, which ignores the correlation between adjacent pixels and cannot mine the overall semantic information of an image. The proposed annotation strategy maps pixels as a word in the vector space and adopts an equivalent embedded mapping for all labels, such that the labels and their corresponding pixels can better describe the contents of remote sensing images in the same low-dimensional vector space.
2. The characterisation of the fused spectral-texture features in the different datasets is stronger than that of the single spectral and texture features. This is because the fused features effectively combine the spectral properties of an image with its texture properties, which represents rich spectral information. The fused features also impart a strong spatial nature. Therefore, the fused features are superior to the single features.
3. The recognition effect is greater for QuickBird remote sensing images than for UVA images. Although the resolution of QuickBird image data is less than that of the UVA images, the spectral band is greater; this enhances the recognition effect for QuickBird images.

#### UC merced land-use data set test results

In order to test whether the End-to-end model is universal and robust in target detection, this experiment was conducted on the public remote sensing data set of UC Merced Land-Use Data Set. The experimental results and analysis are as follows.

Analysis:

From the public data set, 6 types of features were randomly selected and tested using the End-to-end model proposed in this article. It can be seen from the Table 6 that the accuracy rate range is 0.8715–0.9238, the recall rate range is 0.8722–0.8997 and the F value range is 0.8654–0.0.9116 in the 6 types of feature recognition, indicating that the modelalso has good performance when it performs a single location in the UC Merced Land-Use Data Set. The model has good robustness and is suitable for other data sets.

**Table 6:** Experimental results from UC Merced Land-Use Data Set.

End-to-end Model	P	R	F
Airplane	0.8964	0.8722	0.8841
Beach	0.9086	0.8731	0.8904
Forest	0.9157	0.8823	0.8987
River	0.8869	0.8460	0.8660
Harbor	0.8715	0.8595	0.8654
Golfcourse	0.9238	0.8997	0.9116

#### Comparison with conventional deep learning models

**Figure 10:**
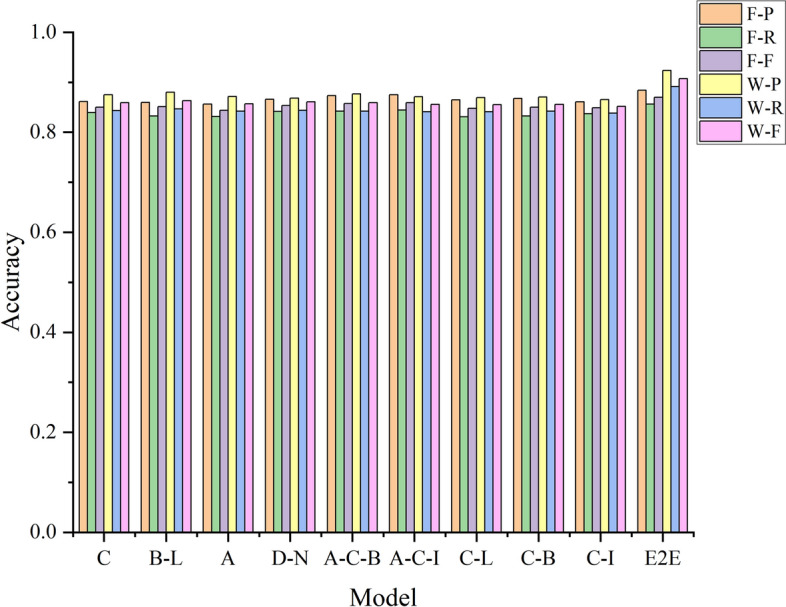
Effect of different deep learning on the evaluation criteria values for UAV images.

**Figure 11:**
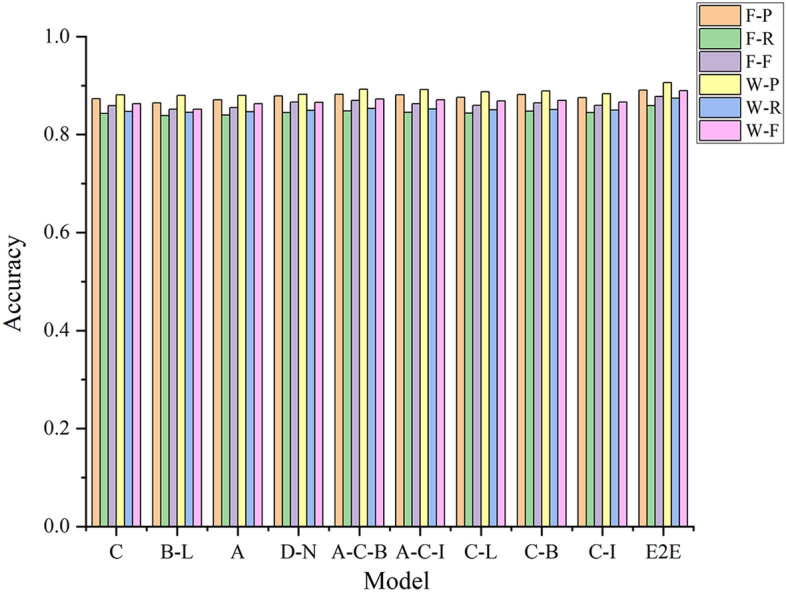
Effect of different deep learning on the evaluation criteria values for Quick Bird images.

The recognition effect and description ability of the proposed method are compared with those of conventional deep learning models using a conventional pixel-by-pixel labelling method and the new labelling strategy method combining word vector features. The experimental F-score results are shown in Fig. 10; P, R and F criteria values are listed in Table 7 for UAV images. Meanwhile, the experimental F-score results for QuickBird images are shown in Fig. 11, whereas all P, R and F criteria values are listed in Table 8.

**Table 7:** Effect of different deep learning models on the evaluation criteria values for UAV images.

Model features		Fusion			Word		
		P	R	F	P	R	F
CNN^25^	C	0.8614	0.8391	0.8501	0.8749	0.8432	0.8588
Bi-LSTM^28^	B-L	0.8597	0.8324	0.8509	0.8802	0.8466	0.8631
Attention^29^	A	0.8564	0.8312	0.8436	0.8713	0.8422	0.8565
Dense Net^31^	D-N	0.8658	0.8414	0.8534	0.8679	0.8435	0.8606
Attention-CNN-Bi-LSTM	A-C-B	0.8732	0.8419	0.8574	0.8766	0.8421	0.8592
Attention-CNN-IndRNN	A-C-I	0.8751	0.8441	0.8593	0.8708	0.8411	0.8557
CNN_LSTM	C-L	0.8649	0.8307	0.8475	0.8692	0.8411	0.8549
CNN-Bi-LSTM	C-B	0.8677	0.8324	0.8497	0.8701	0.8420	0.8558
CNN-IndRNN	C-I	0.8607	0.8369	0.8486	0.8655	0.8382	0.8516
End-to-end	E2E	0.8837	0.8563	0.8698	0.9234	0.8911	0.9069

**Table 8:** Effect of different deep learning models on the evaluation criteria values for Quick Bird images.

Model features		Fusion			Word		
		P	R	F	P	R	F
CNN ^25^	C	0.8732	0.8430	0.8593	0.8809	0.8471	0.8632
Bi-LSTM^28^	B-L	0.8647	0.8388	0.8516	0.8799	0.8452	0.8517
Attention ^29^	A	0.8707	0.8399	0.8550	0.8801	0.8465	0.8629
Dense Net ^31^	D-N	0.8788	0.8451	0.8666	0.8822	0.8496	0.8656
Attention-CNN-Bi-LSTM	A-C-B	0.8820	0.8483	0.8698	0.8926	0.8531	0.8724
Attention-CNN-IndRNN	A-C-I	0.8811	0.8456	0.8630	0.8919	0.8524	0.8707
CNN_LSTM	C-L	0.8761	0.8439	0.8597	0.8872	0.8503	0.8684
CNN-Bi-LSTM	C-B	0.8817	0.8479	0.8645	0.8891	0.8510	0.8696
CNN-IndRNN	C-I	0.8752	0.8446	0.8596	0.8835	0.8502	0.8665
End-to-end	E2E	0.8905	0.8593	0.8778	0.9057	0.8741	0.8896

##### Analysis

1. The description ability and recognition effect of the proposed end-to-end model are superior to those of the conventional deep learning models because the proposed model uses a variety of deep multimodal neural network models to learn the characteristics of an image. For example, the CNN model in the Attention_CNN_Bi-LSTM model can extract the local deep features of an image (i.e. the deep abstract features). The Bi-LSTM network can mine the global semantic features between adjacent pixels in an image and integrate local and global information using the Attention model to highlight key features.
2. The description ability and recognition effect of the Dense network^32^ for the two data sets are greater than those of the CNN and Bi-LSTM network. This is because connections in the Dense network provide integrated information flow while mining deep semantic information, which avoids the loss of detailed information, and realises the reuse of features. However, the CNN and Bi-LSTM network cannot transmit detailed information available at the input layer to the output layer.
3. The end-to-end model further extracts features from the available input feature information and provides connections between pixel word vectors and the global image while reducing the noise to the greatest extent possible. In addition, the end-to-end model correlates Modelling, such that the word vector string of the pixel feature with the greatest probability is obtained during decoding and the image content is accurately described. Therefore, the performance of the end-to-end model is better than the conventional deep learning models investigated.
4. The word vector demonstrates better representation ability than the conventional pixel-by-pixel recognition method for both UAV and QuickBird image data. This is because the proposed model incorporates the new labelling strategy, thereby establishing a deeper relation between image features and labels. Hence, the end-to-end deep learning model provides better descriptions of the complex content in remote sensing images.

## Conclusion

This study proposed an end-to-end image description generation model based on word embedding technology to realise the classification and identification of the species *P. euphratica* and *T. ramosissima* in complex remote sensing images by providing descriptions in precise and concise natural sentences. The proposed method was experimentally verified using QuickBird remote sensing images and UAV images, both of which contain partially fuzzy image attributes. In addition, the description ability and recognition effect of the proposed method were compared with those obtained using conventional deep learning models. The experimental results demonstrated the feasibility of the proposed method. Although the proposed method effectively addresses the fuzzy ambiguity of image classification, the remote sensing images were too fuzzy compared with natural images and the rotational ambiguity was not well resolved. Therefore, addressing the rotational ambiguity and developing a deeper description are our future directions of research.
